# Exploring the Efficacy of a Set of Smart Devices for Postural Awareness for Workers in an Industrial Context: Protocol for a Single-Subject Experimental Design

**DOI:** 10.2196/43637

**Published:** 2023-05-04

**Authors:** Mário Lopes, Susana Lopes, Mariana Monteiro, Mário Rodrigues, Aureliano Fertuzinhos, Augusto de Sousa Coelho, Paulo Matos, Abílio Borges, Teófilo Leite, Cátia Sampaio, Rui Costa, José Alvarelhão

**Affiliations:** 1 School of Health Sciences and Institute of Biomedicine University of Aveiro Aveiro Portugal; 2 School of Health Sciences University of Aveiro Aveiro Portugal; 3 CeNTI – Centre for Nanotechnology and Smart Materials V N Famalicão Portugal; 4 Research Institute for Design, Media and Culture [ID+] School of Design, Management and Production Technologies Northern Aveiro University of Aveiro Aveiro Portugal; 5 ATENA Automação Industrial, Lda Aveiro Portugal; 6 ICC - Indústrias e Comércio de Calçado, SA Guimarães Portugal

**Keywords:** smart wear, smart health, occupational safety, motion sensing, devices, awareness, posture, fatigue, biomechanics, musculoskeletal disorder, smart device, pressure sensing

## Abstract

**Background:**

In manufacturing industries, tasks requiring poor posture, high repetition, and long duration commonly induce fatigue and lead to an increased risk of work-related musculoskeletal disorders. Smart devices assessing biomechanics and providing feedback to the worker for correction may be a successful way to increase postural awareness, reducing fatigue, and work-related musculoskeletal disorders. However, evidence in industrial settings is lacking.

**Objective:**

This study protocol aims to explore the efficacy of a set of smart devices to detect malposture and increase postural awareness, reducing fatigue, and musculoskeletal disorders.

**Methods:**

A longitudinal single-subject experimental design following the ABAB sequence will be developed in a manufacturing industry real context with 5 workers. A repetitive task of screw tightening of 5 screws in a standing position into a piece placed horizontally was selected. Workers will be assessed in 4 moments per shift (10 minutes after the beginning of the shift, 10 minutes before and after the break, and 10 minutes before the end of the shift) in 5 nonconsecutive days. The primary outcomes are fatigue, assessed by electromyography, and musculoskeletal symptoms assessed by the Nordic Musculoskeletal Questionnaire. Secondary outcomes include perceived effort (Borg perceived exertion scale); range of motion of the main joints in the upper body, speed, acceleration, and deceleration assessed by motion analysis; risk stratification of range of motion; and cycle duration in minutes. Structured visual analysis techniques will be conducted to observe the effects of the intervention. Results for each variable of interest will be compared among the different time points of the work shift and longitudinally considering each assessment day as a time point.

**Results:**

Enrollment for the study will start in April 2023. Results are expected to be available still in the first semester of 2023. It is expected that the use of the smart system will reduce malposture, fatigue, and consequently, work-related musculoskeletal pain and disorders.

**Conclusions:**

This proposed study will explore a strategy to increase postural awareness in industrial manufacturing workers who do repetitive tasks, using smart wearables that provide real-time feedback about biomechanics. Results would showcase a novel approach for improving self-awareness of risk for work-related musculoskeletal disorders for these workers providing an evidence base support for the use of such devices.

**International Registered Report Identifier (IRRID):**

PRR1-10.2196/43637

## Introduction

The agenda for decent work, initially promoted by the World Labor Organization, and recently connected to the sustainable development goals of the United Nations [1], includes safety and security at work as one of its strategic axis [2]. The quest to fulfill this axis also reflects the increased demand from consumers regarding concerns about the development of fair and sustainable products. In the same line of concerns, some companies issue bonds incorporating the fulfillment of objectives related to the reduction of accidents at work [3]. Thus, the opportunities offered by the technological developments of the so-called Industry 4.0 could positively influence the indicators related to this set of objectives and concerns, namely with regard to musculoskeletal injuries. Musculoskeletal disorders (MSDs) are the most prevalent work-related health issues and one of the most critical public health problems worldwide [4,5]. Work-related musculoskeletal disorders (WMSDs) are mainly caused by the execution of work tasks and the environment in which work is performed [4]. In the manufacturing industries, tasks requiring excessive force, poor posture, high repetition, and long duration, which induce fatigue and which are stressful, lead to an increased risk of MSDs [4,6,7]. The consequences of WMSDs have a significant and wide-ranging financial impact on both the social and individual levels [8]. In Europe, about 3 of every 5 workers report MSDs. In assembly and manufacturing workers, the most common types of MSDs reported are back pain (55%) and muscular pain in the neck, shoulder, and upper limbs (47%), followed by the lower limbs (33%) [4]. MSDs are responsible for 15% of the total number of (disability-adjusted) life years. Furthermore, more than half (53%) of the workers with MSDs report work absenteeism. Therefore, a high burden on the economy and health care systems is not surprising. In addition, the individual, coworkers, and families are also affected, directly or indirectly, by the injured workers with an associated decrease in physical and mental health and quality of life [9,10].

Due to the high prevalence of musculoskeletal complaints and diseases, and the high associated burden, a significant effort has been made in the prevention of WMSDs [10]. Assessing exposure to risk factors of WMSDs is among the most successful strategies [11]. One of these emerging approaches is wearable devices [11-14]. These are rapidly developing, fostering the promotion of individual healthier lifestyles. Smart workwear is technology embedded into clothing that contacts the skin. This technology can monitor heart rate and movement delivering holistic information on fatigue and body stress. The real-time feedback of data provided can enhance the monitoring of health status, perhaps enabling earlier diagnosis or, in turn, hastening the integration of injury and illness prevention strategies [13,15]. Thus, comfortable smart workwear with movement and posture monitoring seems an adequate solution for the assessment of WMSDs risk and prevention of WMSDs. Although recent advances have been made in posture assessment and monitoring, few studies focus on compensatory adaptations that provide feedback to the user [16,17]. Injury prevention, due to inadequate posture or muscle fatigue, may be engaged with educational information and biofeedback delivered to the operator in real-time conditions or at strategically selected intervals of time during the work shift. Providing individual feedback to the worker may help decrease the exposure of individual workers to ergonomic risk factors and consequently, injury [18].

Thus, this study protocol aims to detect muscle fatigue related to repetitive activities and to explore a smart wear system for correcting biomechanics and reducing fatigue. The system involves the use of a wearable sensorized jacket and shoes and real-time feedback delivered to the worker. This system was created with the aim of preventing the unconscious development of awkward postures, compensatory movements, and fatigue, predisposing to injuries over time. The system will be tested through a single-subject experimental design with human subjects performing a repetitive task in real industry settings. The development of research in a real context on the factory floor raises different challenges. One of these challenges concerns the interference that investigation activities make in the normal production cycle. Thus, the methodological options should consider the least possible impact for the typical activities carried out there. In this sense, within the range of experimental work options, a single-subject experimental design will be able to contribute to these requirements.

## Methods

### Design and Setting

**Figure 1:**
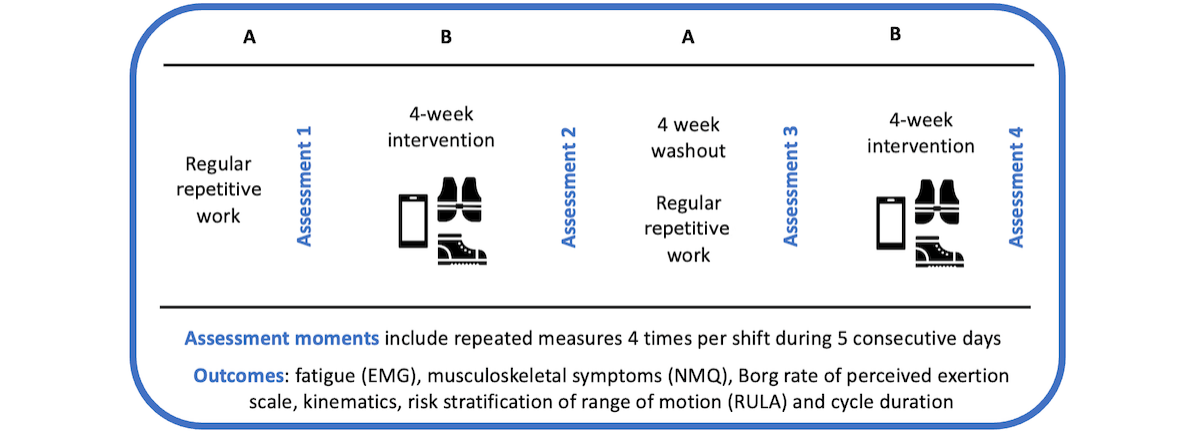
Study flow diagram. EMG: electromyography; NMQ: Nordic Musculoskeletal Questionnaire; RULA: Rapid Upper Limb Assessment.

A longitudinal single-subject experimental design following the ABAB sequence [19] will be developed in a manufacturing industry real context. In the ABAB design, first, a baseline is determined (A), then the intervention is introduced, with the use of the smart wear and real-time feedback to the worker for the first time (B). Next, the intervention is withdrawn, returning to the baseline condition (A). Finally, after baseline stability is reestablished, the intervention is presented (B) for the second time [1,2]. All 4 periods will end with an assessment of the selected outcomes. Figure 1 depicts the study flow. Generally, in this study design, the research is done very thoroughly on 3 to 5 subjects. The interest is always in the meticulous examination of each participant individually, not in the group's average performance. Multiple single-subject experimental studies also present the opportunity to consider not only the individual variables but also analyze the trial at the environmental level [19,20].

### Ethics Approval and Consent to Participate

All participants will give informed consent to participate. All procedures are handled according to the Declaration of Helsinki and regulatory legislation, which includes approval by an ethics committee. The protocol is under review by the Ethics Committee of the University of Aveiro (17-CED/2023). Data will be processed complying with the European General Data Protection Regulation.

### Participants, Eligibility, and Recruitment

This study will include 5 active workers from an industrial manufacturing setting of household appliances in the Region of Aveiro, Portugal. To be included, workers must be aged between 35 and 45 years and have a work experience with the tested workstation for at least 6 months. Exclusion criteria are (1) history of vestibular disorders, (2) neurological, respiratory disease, or spinal surgery, (3) structural spinal problems, (4) medication or a health condition affecting balance, and (5) regular treatments for a musculoskeletal condition.

The workers will be recruited by the occupational health department, which will provide the identification numbers of each selected worker as well as shoe and clothing sizes.

### Study Procedure

A specific task representing a repetitive movement was chosen. The task consists of the vertical screw tightening of 5 screws in a standing position into a piece placed horizontally. A first assessment period will be executed to analyze baseline repetitive work (A) outcomes. Subsequently, the workers will use the smart wearables and be provided with real-time feedback for 4 weeks (B). A second assessment (assessment 2) will be performed to explore the efficacy of the system. Afterward, the workers will have a washout period of 4 weeks to reestablish baseline repetitive work condition (A), followed by assessment 3. Finally, a second 4-week period of intervention (B) and consequent assessment 4, will take place (Figure 1). Each assessment moment will be accomplished in 5 consecutive days, in 4 periods of the day, which includes measurements for each period of 5 cycles of the performed task in the first 10 minutes after starting the shift (to avoid the warm-up period), 10 minutes before the break time, 10 minutes after the break, and 10 minutes before the end of the day's shift. All tests will be performed guaranteeing that in the anterior 48-hour period each individual did not participate in strenuous physical activities, which may affect the selected outcomes.

### Smart Wearable Systems

**Figure 2:**
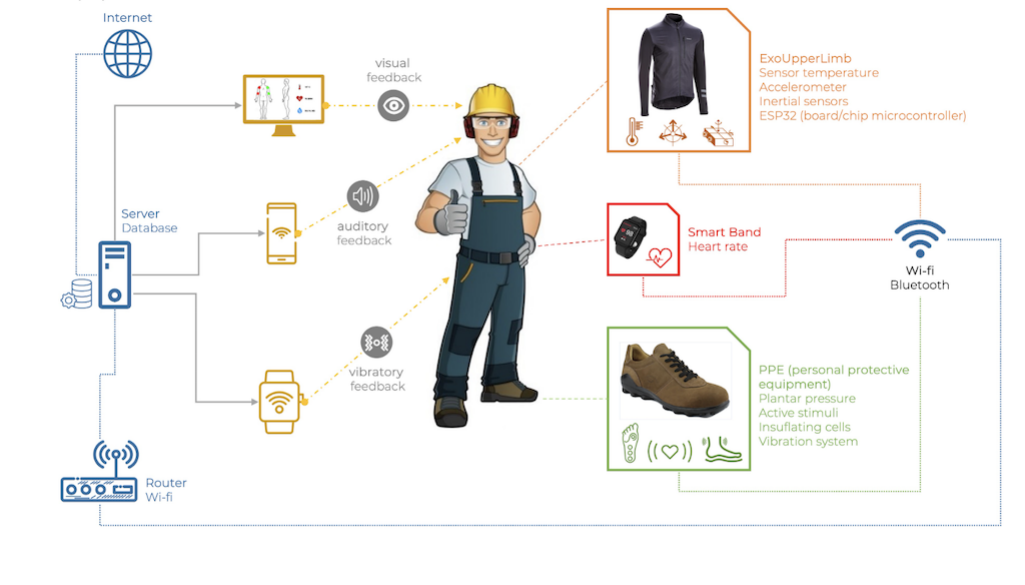
Main system architecture. PPE: Personal Protective Equipment.

The smart wearable systems consist of a sensorized jacket, smart footwear, a smart band, and a feedback system provided to the worker. The sensorized jacket aims to monitor the upper limb (shoulder flexion/extension and abduction/adduction and elbow flexion/extension) and trunk (lumbar flexion/extension) with strategically placed inertial measurement unit (IMU) systems and body temperature with a temperature sensor. Smart footwear consists of a shoe equipped with a set of 6 sensors Force Sensitive Resistors (Sensitronics), to map plantar pressures. The sensors have a 30-mm diameter and work in a 0-300–N force range. They were assembled in a custom substrate printed by RokuPrint (RP 2.2, RokuPrint GmbH). A smart band will monitor heart rate. The main system architecture is presented in Figure 2.

### Data Collection and Outcomes

The primary outcomes are fatigue and musculoskeletal symptoms. Fatigue will be assessed by electromyography (EMG) and musculoskeletal symptoms by the Nordic Musculoskeletal Questionnaire [21].

Secondary outcomes include perceived effort, assessed by the Borg Rating of Perceived Exertion (RPE), range of motion of the main joints in the upper body and trunk, speed, acceleration, and deceleration of movement, through motion analysis, risk stratification of range of motion of the upper limb and trunk based on limits defined in the Rapid Upper Limb Assessment (RULA) [22], and cycle duration in minutes.

For patient characterization, baseline questionnaires including sociodemographic data, physical activity level, quality of life, anxiety and depression, insomnia and sleep quality, fatigue, and pain and disability will be collected. Table 1 displays the assessment flow.

**Table 1:** Assessment flow.

Measurements	Initial measurement	At the start of the shift	During the shift	After the end of the shift
Sociodemographic information and habits	✓			
Quality of life—EUROHIS-QOL-8	✓			
Musculoskeletal pain (NMQ^a^) and adapted NMQ	✓	✓		✓
Physical activity (RAPA^b^)	✓			
Anxiety (GAD-7^c^) and depression (PHQ-9^d^)	✓			
Subjective fatigue (FACIT-F^e^)	✓			
Sleep quality (BaSIQS^f^)	✓			
Heart rate			✓	
Joint range of motion			✓	
Ergonomic risk (RULA^g^)			✓	
Electromyography			✓	
Perceived exertion (RPE^h^)		✓	✓	✓

^a^NMQ: Nordic Musculoskeletal Questionnaire.

^b^RAPA: Rapid Assessment of Physical Activity questionnaire.

^c^GAD-7: General Anxiety Disorder 7-item scale

^d^PHQ-9: Patient Health Questionnaire-9.

^e^FACIT-F: Functional Assessment of Chronic Illness Therapy—Fatigue.

^f^BaSIQS: Basic scale on insomnia complaints and quality of sleep questionnaire.

^g^RULA: rapid upper limb assessment.

^h^RPE: rate of perceived exertion.

### Instruments

#### Electromyography

EMG recording will be conducted using the Cometa EMG wireless portable system (Covidien Kendall) with disposable silver 24-mm surface electrodes. Electrode application will proceed following the recommended procedures of skin preparation: shaving of the area; rubbing and cleaning with alcohol; following a 10-minute resting time to reduce skin impedance [23]. The EMG electrodes will be placed according to manufacturer’s recommendations on the upper trapezius, posterior deltoid, triceps medial, biceps brachii, the lumbar portion of the erector, biceps femoris, tibialis anterior, and gastrocnemius. All trials will be visually examined for signal quality and measurement errors. Muscle activity data will be collected with a sampling rate of 2000 Hz. EMG data will be processed with a customized MATLAB script (R2017b, MathWorks), where a fourth-order Butterworth band-pass filter from 10 to 500 Hz will be applied to minimize the appearance of electromyographic artifacts [24]. EMG signal will be normalized using a method that divides each point that constitutes the processed EMG by the peak value acquired from the same EMG. The root-mean-square of the EMG data and the averaged rectified value will be calculated to determine the signal amplitudes. Muscle fatigue will be characterized by median frequency and amplitude.

#### Motion Analysis

A 3D biomechanical motion analysis for kinetics and kinematics parameters will be recorded with the Vicon-Nexus 3D motion capture system (Vicon Vero, v2.2). Reflective markers (clusters) will be placed on the midpoint between the left shoulder and left elbow, the midpoint between right shoulder and right elbow, the midpoint between the left elbow and left wrist, the midpoint between the right elbow and right wrist, and seventh cervical vertebra and sacral region (midpoint between right posterior superior iliac and left posterior superior iliac) [25]. Additional markers will be placed on the screwdriver (top of the shaft) by the same experienced examiner. 3D marker trajectory data will be collected at 100 Hz with 6 cameras. Before starting the calibration, all reflective objects will be removed from the workspace so that they do not interfere with the recording. Necessary care will be taken to warrant that the worker’s clothing is also nonreflective [26]. Prior to the assessment of the subjects, the workstation will be prepared following the recommended procedures of calibration of both optical and video reference cameras according to the manufacturer's recommendations manual. The kinematic variables of interest are the range of motion of the neck, trunk, shoulder and elbow, speed, acceleration, and deceleration. The range of motion will be calculated as the maximum minus minimum joint angle in each selected sample.

### Questionnaires

#### Overview

For all the questionnaires, the Portuguese-validated version will be used.

#### Musculoskeletal Symptoms

To assess MSDs and symptoms associated with the working population, the Nordic Musculoskeletal Questionnaire will be used [21]. This questionnaire encompasses 27 questions (3 questions related to the 9 anatomical regions: neck, shoulders, wrists/hands, upper back, low back, hips/thighs, knees, and ankles/feet) with a binary response (yes or no) and a question to rate pain on a numerical scale [21]. This questionnaire presents a good test-retest reliability and good internal consistency [21]. To assess musculoskeletal symptoms before and after the shift on evaluation days, only the first question will be used and adapted for the recall period; instead of the last 12 months, a period of the last 8 hours will be considered.

#### Perceived Exertion

The Borg RPE scale is a tool for measuring an individual’s effort and exertion, breathlessness, and fatigue during physical work and so is highly relevant for occupational health and safety practice. In this scale, 6 corresponds to “No Effort” and 20 corresponds to “Extremely Hard” [27].

#### Ergonomic Risk

The RULA was selected to assess ergonomic risk in this study. This tool was developed to assess workers’ exposure to ergonomic risk work associated with musculoskeletal injuries of the upper limbs, taking into consideration biomechanical and postural load requirements of work task demands on the neck, trunk, and upper limbs. This instrument provides a simple scoring method based on the range of motion, which can identify joints at risk. The output of the RULA assessment tool is the final RULA score, which is a single score that represents the level of MSD risk for the job task being evaluated, ranging from a minimum of 1 to a maximum of 7 points [22].

#### Physical Activity Level

The level of physical activity will be assessed with the Rapid Assessment of Physical Activity questionnaire (RAPA). This version presents good internal and construct validity [28]. The RAPA evaluates the level and intensity of leisure-time physical activity, as well as strength and flexibility training in a 9-item, self-administered questionnaire [28].

#### Quality of Life

Participants' quality of life will be assessed with the Portuguese version of the EUROHIS-QOL 8-item index questionnaire [29]. This questionnaire presents a good internal consistency, test-retest reliability as well as construct, discriminant, and convergent validity [29]. In this quality-of-life measure, each of the domains (physical, psychological, social relationships, and environment) is represented by 2 items. The result is a global index, calculated from the sum of the 8 items, with a higher value corresponding to a better perception of quality of life [30].

#### Depression and Anxiety Symptoms

The Patient Health Questionnaire-9 (PHQ-9) will be used to assess signs of depression [31]. This tool reports to the last 2 weeks and consists of a depression module with 9 items, using a 4-point response scale, ranging from “0” (not at all) to “3” (nearly every day). The PHQ-9 presents good internal consistency, convergent validity, and criterion validity. Total scores of 5, 10, 15, and 20 represent cut points for mild, moderate, moderately severe, and severe depression, respectively [32]. Anxiety-related signs and their severity will be assessed by the General Anxiety Disorder 7-item scale (GAD-7) [33]. The GAD-7 index is obtained by adding the scores from the questionnaire, after having assigned 0 to 3, depending on the severity. The cutoff points 5, 10, and 15 classify the anxiety as none/normal (0-4), mild (5-9), moderate (10-14), and severe (15-21). In general, anyone who scores 8 or above can be considered as having significant anxiety symptoms [34]. This scale presents cultural adaptation, excellent internal consistency, internal validity, and very good reliability [33].

#### Sleep Quality

The Basic Scale on Insomnia Complaints and Quality of Sleep (ie, BaSIQS) questionnaire is based on a set of 7 questions assessing nocturnal experiences in terms of subjective sleep quality and symptoms of insomnia [35]. Except for the final 2 items, which are scored on a reversed 5-point Likert scale, all items are scored on a scale of 0 to 5, with higher scores indicating poorer sleep. The results on reliability, validity, and accuracy support the use of this questionnaire for research and practical purposes [35].

#### Subjective Fatigue

Fatigue will be assessed with the Functional Assessment of Chronic Illness Therapy- Fatigue. This 13-item tool measures an individual’s level of fatigue during their usual daily activities over the past week. Scoring is based on a 4-point Likert scale (4=not at all fatigued to 0=very much fatigued), with a ranging total score of 0 to 52 (0=worst possible score; 52=best possible score indicating no fatigue) [36]. This questionnaire presents high internal consistency, good convergent and divergent validity, and satisfactory test-retest reliability [37].

### Data Management and Monitoring

The collection and data storage will be made in digital format, including those from the questionnaires. The entered data will be checked for completeness, validity, and plausibility by a member of the research team.

### Data Analysis

Structured visual analysis techniques will be conducted to observe the effects of the intervention using a Single-Case Visual Analysis package for R. Graphical (RStudio) representations of data for each participant will be plotted considering primary and secondary outcomes on the y-axis and time on the x-axis. The results for each variable of interest will be compared in-between the different time points of the work shift and longitudinally considering each assessment day as a time point.

For each outcome (primary or secondary) and each moment mean, median, range, and stability envelope will be calculated. The level of change and difference between the first and last values of each period will be compared. The next step includes the calculation trend using the split middle method of trend estimation. After this procedure, percent of data points within the stability envelope (80% of data points between ±25% of median) will be considered. The final step is using the “freehand method” to evaluate data path [38].

## Results

Enrollment for the study will start in April 2023. We plan to complete the post assessments of the last worker in April 2023. The results are expected to be available still in the first semester of 2023.

## Discussion

### Significance of the Study

Given the high prevalence of WMSDs and the associated economic and personal burden, preventive measures such as smart wear for monitoring and providing feedback are crucial. The smart wearables analyzed under this study aim to create awareness of WMSDs risk and empower the worker to reduce the associated risk and have a healthier work environment and higher quality of life. Investing in preventive measures is especially rewarding, since workers in countries and sectors where more preventive measures are in place are less likely to report MSD complaints [10]. Consequently, preventive measures result in an increase in economic benefits for the industries with greater production and worker adherence to these jobs.

The proposed study is, to our best knowledge, one of the first to use devices that provides real-time feedback to industrial workers about posture. This real-time feedback will include the body location at risk, which can lead to the correction of body biomechanics. Posture and movement monitoring technology can help detect and subsequently reduce the compensatory movement patterns, promoting a decrease in fatigue and musculoskeletal symptoms.

### Strengths and Limitations

This study is a single-case experimental design. This method design was primarily developed to assess individual behavior. Given the main goal is to develop and test smart wear for reducing fatigue and poor biomechanics, a single-subject approach seems adequate. This design enables the possibility of rapidly determining if the wearables are effective. Immediate changes can be observed firsthand, quickly, and in individual participants. Consequently, adjustments can be rapidly made, if necessary. Contrary to the single-subject approach, a large sample statistical approach will require weeks or months of participant testing, mean calculations, and statistical analyses before the effectiveness may be determined. Conceptually, the treatment condition must influence individual behavior and assure both research intra- and interparticipant replication. Therefore, an ABAB design will be executed, assuring both intra- and interparticipant intervention and control [38]. Graphical analysis of the variability of each outcome, in each of the repeated measures in time, will be performed to explore the possible indicators of fatigue when performing the selected activity.

### Conclusions

This proposed study will explore a strategy for reducing fatigue and musculoskeletal symptoms in industrial workers, possibly preventing WMSDs, using smart wearables that provide real-time feedback about biomechanics. Results would showcase a novel approach for improving postural awareness and self-awareness of risk for WMSD providing an evidence-based support for the use of such devices. Further research could explore other health indicators and individualized approaches to prevent similar health risks.

## Abbreviations

EMG: electromyography
GAD-7: General Anxiety Disorder 7-item scale
MSD: musculoskeletal disorder
PHQ-9: Patient Health Questionnaire-9
RAPA: Rapid Assessment of Physical Activity questionnaire
RPE: rating of perceived exertion
RULA: risk stratification of range of motion
WMSD: work-related musculoskeletal disorder

## Data Availability

The data sets generated during and analyzed during the current study are available from the corresponding author on reasonable request.
